# Altered expression of caspases-4 and -5 during inflammatory bowel disease and colorectal cancer: Diagnostic and therapeutic potential

**DOI:** 10.1111/cei.12617

**Published:** 2015-05-06

**Authors:** B Flood, K Oficjalska, D Laukens, J Fay, A O'Grady, F Caiazza, Z Heetun, K H G Mills, K Sheahan, E J Ryan, G A Doherty, E Kay, E M Creagh

**Affiliations:** * School of Biochemistry and Immunology, Trinity Biomedical Sciences Institute, Trinity College DublinIreland; † Department of Gastroenterology, Ghent UniversityGhent, Belgium; ‡ Pathology Department, RCSI and Beaumont HospitalDublin; § Centre for Colorectal Disease, St Vincent's University Hospital and School of Medicine and Medical Sciences, University College DublinIreland

**Keywords:** caspases, colorectal cancer, inflammation, inflammatory bowel disease

## Abstract

Caspases are a group of proteolytic enzymes involved in the co-ordination of cellular processes, including cellular homeostasis, inflammation and apoptosis. Altered activity of caspases, particularly caspase-1, has been implicated in the development of intestinal diseases, such as inflammatory bowel disease (IBD) and colorectal cancer (CRC). However, the involvement of two related inflammatory caspase members, caspases-4 and -5, during intestinal homeostasis and disease has not yet been established. This study demonstrates that caspases-4 and -5 are involved in IBD-associated intestinal inflammation. Furthermore, we found a clear correlation between stromal caspase-4 and -5 expression levels, inflammation and disease activity in ulcerative colitis patients. Deregulated intestinal inflammation in IBD patients is associated with an increased risk of developing CRC. We found robust expression of caspases-4 and -5 within intestinal epithelial cells, exclusively within neoplastic tissue, of colorectal tumours. An examination of adjacent normal, inflamed and tumour tissue from patients with colitis-associated CRC confirmed that stromal expression of caspases-4 and -5 is increased in inflamed and dysplastic tissue, while epithelial expression is restricted to neoplastic tissue. In addition to identifying caspases-4 and -5 as potential targets for limiting intestinal inflammation, this study has identified epithelial-expressed caspases-4 and -5 as biomarkers with diagnostic and therapeutic potential in CRC.

## Introduction

Inflammatory bowel disease (IBD) is characterized by chronic, recurrent inflammation of the gastrointestinal tract and has two main forms – Crohn's disease (CD) and ulcerative colitis (UC). The prevalence of IBD in Western countries is estimated at one in 1000 inhabitants 1, with worldwide prevalence increasing during the last 50 years 2. Numerous studies have consistently supported the oncogenic impact of chronic inflammation on colorectal mucosa; thus, IBD patients have an associated risk of developing colorectal cancer (CRC), with a cumulative 30-year risk of UC-associated CRC of close to 20% 3–5. CRC represents the second most common incident solid organ cancer in females (after breast cancer) and males (after prostate cancer), with an estimated annual incidence in 2008 of 1·2 million worldwide 6. The pathogenesis of CRC is complex and is influenced by genetics, lifestyle and dietary factors; however, an inflammatory microenvironment is a critical component for CRC progression, regardless of the initiation pathway 7,8.

In humans, caspases-1, -4 and -5 constitute the inflammatory caspase subfamily with roles in both inflammation and cell death 9,10. Excessive production of the inflammatory cytokines, interleukin (IL)-1β and IL-18, which are dependent upon inflammasome-mediated caspase-1 activation, is found in inflamed colons of IBD patients 11,12. IL-1β expression is also up-regulated during CRC and has been identified as a key factor in tumour progression and metastasis 13,14. Thus, it is likely that caspase-1 has a role in driving intestinal inflammation during IBD and CRC 15. The roles of caspases-4 and -5 during inflammation are less well characterized than caspase-1, due to the fact that there is no direct homologue of caspases-4 or -5 in the mouse. However, murine caspase-11 is considered to be the functional orthologue of caspases-4 and -5, which are thought to have arisen following a gene duplication event 16. To date, no physiological substrates of caspases-4 or -5 have been verified *in vivo*, although there is a growing body of evidence to support their proinflammatory involvement in a non-canonical inflammasome. Caspase-4 has been implicated in NLRP3 [neuronal apoptosis inhibitory protein, leucine-rich repeat (LRR) and pyrin (PYD) domains-containing protein 3] inflammasome activation in keratinocytes following ultraviolet B (UVB) irradiation 17, and transgenic mice expressing human caspase-4 display higher endotoxin sensitivity, with bone marrow-derived macrophages (BMDMs) from these mice producing mature IL-1β and IL-18 following Toll-like receptors (TLR)-2 and -4 priming alone 18. Caspase-5 was identified originally as a component of the NLRP1 inflammasome 19. However, the majority of studies surrounding non-canonical inflammasome activation have been carried out in murine systems examining caspase-11 10.

A number of mutational analysis studies have been carried out to determine whether or not alterations in inflammatory caspase genes may be involved in the development of human cancers 20,21. These have revealed that the overall incidence of solid tumour mutations is rare for caspases-1 and -4 and occasional for caspase-5 20–22, suggesting that mutations in inflammatory caspases are not implicated causally in oncogenesis 23. Our study examines the expression, rather than the sequence, of inflammatory caspases in tissue from IBD and CRC patients. The revelation that murine caspase-11 is involved in regulating IL-1β production through a non-canonical inflammasome following Gram-negative bacterial infection 24,25 has led us to investigate whether the functional homologues of caspase-11 and human caspases-4 and -5 24,26 may be involved in mediating intestinal inflammation during IBD and CRC.

## Methods

### Clinical samples

#### Belgian IBD patient population

This study was approved by the regional ethics committee (EC 2000/242), and all participating patients signed informed consent. In total, 103 biopsies were retrieved from controls [*n* = 23; 10 males, 13 females, average age 51 yeras (range = 22–69 years)] and patients with CD [*n* = 54; 27 males, 27 females, average age 37 years (range = 8–72)] or UC [*n* = 16; eight males, eight females, average age 38 years (range = 7–61)]. All patients within this cohort were medication-free or on 5-ASA. In addition, the majority of patients have been included previously in a genome-wide scan 27.

#### Irish UC patient population

This study was approved by the Research and Ethics committee of St Vincent's University Hospital. All subjects provided written informed consent. Intestinal biopsies were retrieved from 36 patients with a diagnosis of UC, with prospective Mayo scores of endoscopic activity: Mayo 0 [inactive, *n* = 11; three males, eight females, average age 46 years (range = 19–60 years)], Mayo 1 [minimal, *n* = 7; six males, one female, average age 41 years (range 34–64 years)], Mayo 2 [mild, *n* = 8; eight males, average age 37 years (range = 22–76 years)] and Mayo 3 [moderate to severe, *n* = 10; seven males, three females, average age 40 years (range = 29–61 years)]. All but four patients in this cohort were receiving medication, including aminosalicylates, corticosteroids, immunomodulators or biological therapy (adalimumab or infliximab), according to best clinical practice for treatment of their disease.

#### Biopsy explant cultures

Colonic biopsy specimens were obtained from patients undergoing colonoscopy either for UC or for surveillance investigation (controls). Four patients were recruited prior to endoscopy and informed consent was obtained. At the time of endoscopy, two to four biopsies were taken from healthy, non-adjacent normal and mild inflamed tissue, and four to eight biopsies were taken from severe inflamed tissue. Tissue fragments were placed directly into ice-cold buffer [phosphate-buffered saline (PBS) containing 100 U/ml penicillin, 100 µg/ml streptomycin, 4 µg/ml fungizone, 30 µg/ml gentamicin (Invitrogen, Carlsbad, CA, USA)] and washed three times before dividing into equal (∼5 mm) sections and transferring into 96-well flat-bottomed plates (one section per well). Colon sections were covered with media [RPMI with penicillin/streptomycin and 20% fetal bovine serum (FBS) (Invitrogen)] supplemented with caspase inhibitor Z-YVAD-FMK [Z-Tyr-Val-Ala-Asp(OMe)-fluoromethylketone] (R&D Systems, Minneapolis, MN, USA) or vehicle control [dimethylsulphoxide (DMSO)]. Samples were incubated at 37 °C for 18 h before harvesting supernatants and assaying for IL-1β and IL-6 production [eBiosciences/Biolegend (San Diego, CA, USA) enzyme-linked immunosorbent assay (ELISA) kits], according to the manufacturer's guidelines. Colonic biopsies were homogenized in radioimmunoprecipitation assay (RIPA) buffer [50 mM Tris, pH 8, 150 mM NaCl, 0·1% (w/v) sodium dodecyl sulphide (SDS), 0·5% (w/v) sodium deoxycholate and 1% (v/v) NP-40] supplemented with protease inhibitors (Sigma, St Louis, MO, USA). Ten μg protein lysate was analysed by Western blot for expression of capase-1 (Santa Cruz Biotechnologies, Santa Cruz, CA, USA), caspase-4 and -5 (MBL) and β-actin (Sigma).

#### Irish CRC, IBD-associated CRC and polyp patient populations

The protocol was approved by the Medical Research Ethics Committee, Beaumont hospital. Areas of tumour and adjacent normal tissue from 25 sporadic CRC patients (see Table1 for characteristics) were assessed by immunohistochemistry (IHC) for inflammatory caspases-1, -4 and -5 expression levels. Explant tissue from a further eight patients with IBD-associated CRC [six males, two females, average age 55 years (range = 36–76)] were assessed for inflammatory caspase expression in areas of tumour, inflammation and normal adjacent tissue. Polyp tissue sections were assessed from five patients presenting with colon polyps and analysed for inflammatory caspase-4 expression in areas of low-grade dysplasia (LGD) and normal adjacent tissue.

**Table 1:** Characteristics of colorectal cancer (CRC) patients and tumours examined for caspases-1, 4 and -5 expression by immunohistochemistry (IHC)

Patient no.	Gender, female (F), male (M)	Age	Location	Type	Stage
1	F	76	Right hemicolectomy	Invasive moderately differentiated adenocarcinoma	pT3 N2 Mx Duke's C
2	M	51	Anterior resection	Moderately differentiated adenocarcinoma	pT3 N1 Mx Duke's C
3	F	78	Right hemicolectomy	Invasive moderately differentiated adenocarcinoma	pT3 N0 Mx Duke's B
4	F	71	Anterior resection	Invasive moderately differentiated adenocarcinoma	pT4 N2 Mx Duke's C
5	M	78	Right hemicolectomy	Villius adenomata	pTis N0 Mx
6	F	41	Anterior resection	Focally invasive moderately differentiated adenocarcinoma, post-neoadjuvant chemotherapy	ypT3 N0 Duke's B
7	M	61	Sigmoid	Invasive moderately differentiated adenocarcinoma	pT3 N0 Mx Duke's B
8	F	78	Right hemicolectomy	Invasive moderately differentiated adenocarcinoma	pT2 N0 Mx Duke's A
9	M	64	Rectum	Invasive moderately differentiated adenocarcinoma	pT2 N2 Mx Duke's C
10	M	59	Rectum	Invasive moderately differentiated adenocarcinoma, post-neoadjuvant chemotherapy	ypT3 N0 Duke's B
11	F	50	Right hemicolectomy	Ulcerated moderately differentiated adenocarcinoma	pT3 N0 Mx
12	M	74	Sigmoid	Moderately differentiated adenocarcinoma	pT3 N1 Mx Duke's C
13	F	62	Anterior resection	Infiltrating moderately differentiated adenocarcinoma	pT1 N0 Duke's A
14	F	44	Recto-sigmoid	Moderately differentiated adenocarcinoma	pT2 N0 Mx Duke's A
15	F	39	Proctocolectomy	Synchronous adenocarcinoma in familial adenomatous polyposis	pT3 N1 Mx Duke's C
16	M	64	Right hemicolectomy	Invasive moderately differentiated mucinous adenocarcinoma	pT3 N2 Mx Duke's C
17	F	53	Right hemicolectomy	Invasive moderately differentiated adenocarcinoma	pT3 N0 Mx Duke's B
18	F	65	Right hemicolectomy	Invasive moderately differentiated adenocarcinoma	pT3 N0 M1
19	F	72	Sigmoid	Invasive moderately differentiated adenocarcinoma	pT1 N0 Mx
20	F	81	Rectum	Moderately differentiated adenocarcinoma	pT1 N0 Mx
21	F	69	Right hemicolectomy	Moderately differentiated adenocarcinoma	pT3 N1 Mx Duke's C
22	F	78	Colon (no area specified)	Ulcerated moderately differentiated adenocarcinoma	pT4 N0
23	F	81	Colon (no area specified)	Well-differentiated adenocarcinoma	pT3 N1 Mx Duke's C
24	M	83	Anterior resection	Moderately differentiated adenocarcinoma	pT3 N2
25	M	70	Rectosigmoid	Poorly differentiated adenocarcinoma, post-neoadjuvant chemotherapy	ypT3 N0 Mx

### RNA isolation and conversion to cDNA

Total RNA was extracted from biopsies using the Qiagen RNeasy Mini Kit (Qiagen, Valencia, CA, USA), with on-column DNAse treatment according to the manufacturer's instructions. The quality and concentration of RNA were assessed by subjecting samples to automated gel electrophoresis and Experion analysis (Bio-Rad, Hercules, CA, USA). Samples with a 28S/18S ratio between 1·6 and 1·8 and a minimum RNA quality index of 7 were included in the analysis. The WT-Ovation™ system from NuGEN (NuGEN Technologies Inc., San Carlo, CA, USA) was used to prepare and amplify cDNA, starting from 50 ng of total RNA, according to the manufacturer's instructions.

### Quantitative polymerase chain reaction (qPCR)

The real-time PCR primer and probe database (RTprimerDB) was used for primer design and *in-silico* primer evaluation [absence of secondary structures, single nucleotide polymorphisms (SNPs) and aspecific binding using the Basic Local Alignment Search Tool (BLAST)]. The conditions of qPCR were set to 60 °C annealing temperature, 50 mM Na^+^ and 3 mM Mg^2+^. To test the PCR-efficiency of a primer set a dilution series of cDNA or gDNA was subjected to qPCR analysis, and the efficiency was calculated as (10^−1/slope ^− 1) × 100. Primer sets with efficiencies of 90–110% were used. Primer sequences used were as follows – CASP1 forward: TGCCTGTTCCTGTGATGTGGAGGA, CASP1 reverse: CAGTGGTGGGCATCTGCGCT; CASP4 forward: ACCGTGGGGAACTGTGGGTCA, CASP4 reverse: CCAGGACACGTTGTGTGGCGT; and CASP5 forward: CGCAGACGCCTGGCTCTCAT, CASP5 reverse: AGCCCAGGCCTTGAAGCAGC. SYBR green I containing PCR mixtures (8 μl reaction volume; Roche, Basel, Switzerland) was run on a Lightcycler in 384-well format (LC480; Roche). Cycling conditions were 10 min at 95 °C and 40 cycles at 95 °C for 15 s and 60 °C for 60 s. For each primer set, melting-peak analysis was performed to verify the specificity of the reaction in each well. Expression data were normalized using the median expression of succinate dehydrogenase complex subunit A (SDHA), hypoxanthine phosphoribosyltransferase (HPRT) and glyceraldehyde-3-phosphate dehydrogenase (GAPDH) as reference genes 28. Data analyses were performed using the accompanied software and results were imported to GraphPad Prism (GraphPad Software, San Diego, CA, USA) for further analysis.

### Immunohistochemistry of UC patient biopsies

Immunohistochemistry was performed using formalin-fixed, paraffin-embedded (FFPE) tissues obtained from UC patients. A routine three-stage immunoperoxidase labelling technique incorporating avidin–biotin immunoperoxidase complex (Dako, Glostrup, Denmark) was used. Sections were incubated with primary anti-caspase-1 (Santa Cruz Biotechnologies Inc.), anti-caspases-4 and -5 (Medical and Biological Laboratories (MBL), Woburn, MA, USA) antibodies for 1 h. Sections were also incubated with an appropriate isotype-matched mouse/rabbit monoclonal antibody as a negative control. Diaminobenzadine tetrahydrochloride (DAB; Sigma) was used to visualize staining. Images were captured using the Olympus DP50 light microscope and AnalySIS software (Soft Imaging System Corporation, Lakewood, CO, USA). Caspase expression was assessed by two blinded reviewers using a validated semiquantitative scoring method. All IHC-stained cells were assessed by a combined score of intensity and percentage of nuclear and cytoplasmic staining. Staining intensity was graded using a scale of 0–3, where 0 = negative, 1 = weak, 2 = moderate and 3 = strong. Percentage positivity was graded using a scale of 0–4, where 0 = no stained cells, 1 = 1–25% stained cells, 2 = 25–50% stained cells, 3 =50–75% stained cells and 4 = 75–100% stained cells.

### CRC immunohistochemical analysis

Inflammatory caspase IHC analysis was carried out on a Bond-III immunostainer from Leica Biosystems (Newcastle upon Tyne, UK). The Bond-III system dewaxed slides before pretreatment with Bond Epitope Retrieval Solution I. Primary antibodies – anti-Casp-4 (MBL); anti-Casp-5 (MBL) and anti-Casp-1 (Santa Cruz Biotechnologies Inc. – were diluted in Bond primary antibody diluent. Detection and visualization of stained cells was achieved using the Bond Polymer Refine Detection Kit, using DAB as the chromagen. Tissues were counterstained with haematoxylin and coverslipped. Appropriate negative controls (omission of primary antibodies) were used in all assays.

### Statistical analysis

Values are expressed as the median and range or mean ± standard error of the mean (s.e.m.). The software package GraphPad Prism, version 5 (GraphPad Software) was used to perform statistical analysis. Differences in parameters between two groups were performed using the non-parametric Kruskal–Wallis test (with Dunn's *post-hoc* comparison to control) for qPCR analysis and unpaired Mann–Whitney *U*-tests for IHC expression data. Correlation analysis was undertaken by calculating the Spearman's correlation coefficient. *P-*values of less than 0·05 were considered statistically significant.

## Results

**Figure 1:**
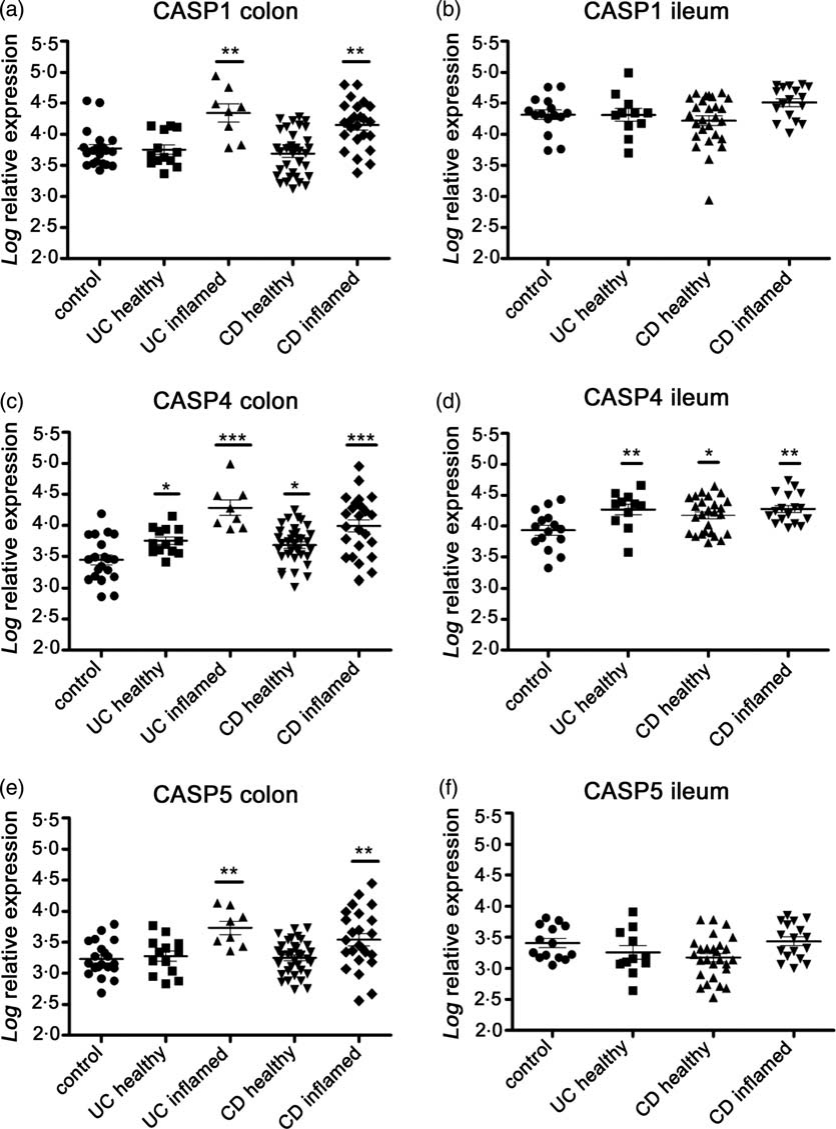
Increased inflammatory caspase gene expression in inflammatory bowel disease (IBD) patient biopsies from a Belgian cohort. Relative expression of (a,b) caspase-1, (c,d) caspase-4 and (e,f) caspase-5 in biopsy specimens from non-IBD healthy control (*n* = 21), active ulcerative colitis (UC) patients in endoscopically healthy (*n* = 10) and inflamed (*n* = 11) areas, and active Crohn's disease (CD) patients in healthy (*n* = 38) and inflamed (*n* = 21) colon and ileum as quantified by quantitative polymerase chain reaction (qPCR). Data are normalized to the median of three stably expressed reference genes and represent median and range; *P < 0·05; **P < 0·01; ***P < 0·001 (Kruskal–Wallis test with Dunn's *post-hoc* comparison to control).

A study was designed to examine the extent of inflammatory caspase expression in IBD patient intestinal biopsies. We used real-time PCR to amplify and quantify *CASP1*, *CASP4* and *CASP5* from cDNA prepared from 103 biopsies of 53 CD patients, 26 UC patients and 24 healthy patients. UC/CD healthy or inflamed tissue was obtained from UC/CD patients with and without endoscopic evidence of inflammation and control tissue was obtained from endoscopically normal biopsies from non-IBD control individuals. Caspase-1 expression was elevated significantly in colonic tissue samples from inflamed UC/CD patients (Fig. 1a). Caspases-4 and -5 expression was also elevated significantly in inflamed colonic tissue, suggesting that, in addition to caspase-1, caspases-4 and -5 may also have a role during intestinal inflammation (Fig. 1c,e). The data shown in Fig. 1c,d revealed that caspase-4 expression is higher in non-inflamed tissue from IBD patients (in both ileum and colon), suggesting that it may be an early marker of IBD. In contrast, both caspases-1 and -5 expression was significantly higher in inflamed colonic, not ileal, tissue (Fig. 1a,b,e,f), suggesting their involvement with colitis, rather than ileitis. Taken together, these data support the hypothesis that inflammatory caspases-4 and -5 have an important role in intestinal inflammation observed during IBD.

**Figure 2:**
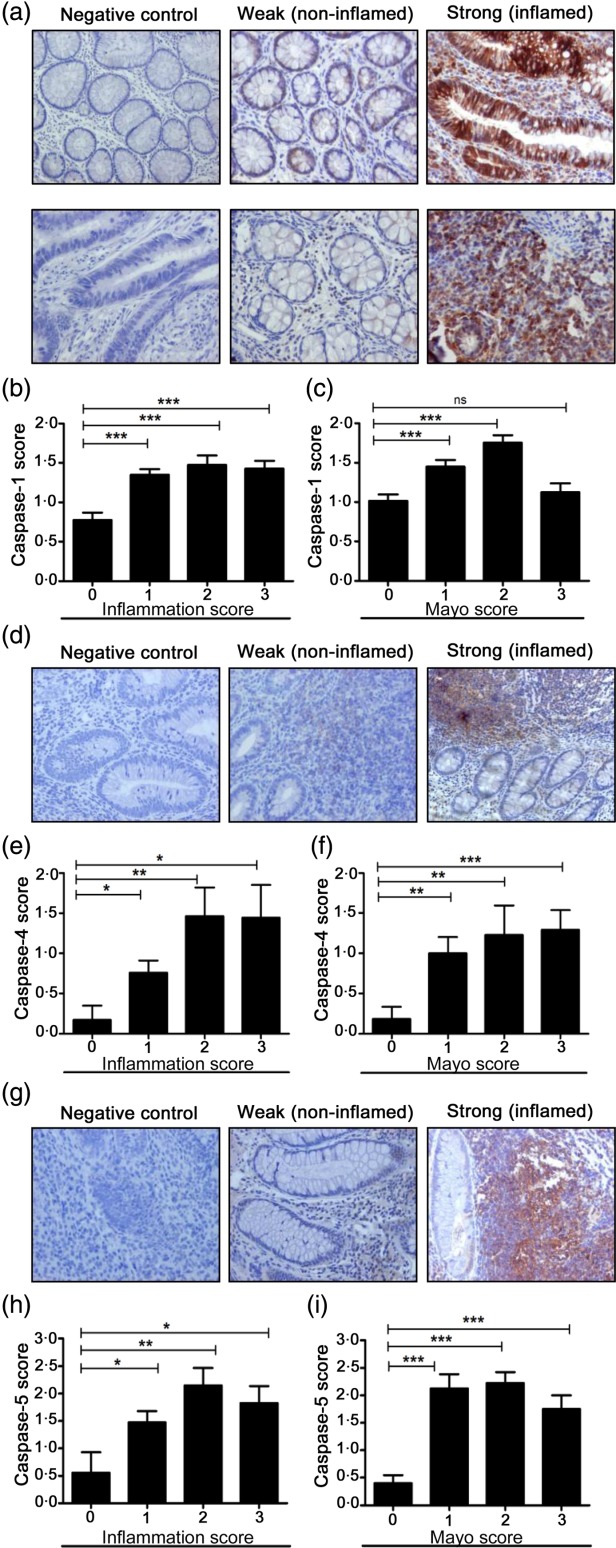
Inflammatory caspases-4 and -5 expression correlates with clinical disease and inflammation scores in ulcerative colitis (UC) patient biopsies from an Irish cohort. (a) Representative images of caspase-1 expression pattern in epithelial cells (top panel) and stromal cells (bottom panel) from non-inflamed and inflamed UC biopsy sections. Negative staining was observed for isotype rabbit/mouse immunoglobulin (Ig)G control (negative control). Original magnification ×20. (d,g) Representative images of caspases-4 and -5 expression patterns in stromal cells of UC biopsy sections. (b,e,h) Caspases-1, -4 and -5 expression levels correlate with histological inflammatory scores for UC colonic biopsies. Inflammation score was graded on a scale of 0–3 (with (0) indicating no activity, (1) mild activity, (2) moderate activity and (3) severe activity. (c,f,i) Caspase-1, -4 and -5 expression levels *versus* endoscopic Mayo score. Immunohistochemistry (IHC) was performed on colon tissue from 11 non-inflamed UC patients and 25 patients with varying degrees of inflammation. Data represent mean ± standard error of the mean (s.e.m.); *P < 0·05; **P < 0·01; ***P < 0·001 (Mann–Whitney *U*-test).

To confirm this hypothesis, we examined the relationship between inflammatory caspase expression and both inflammation and disease activity in UC patients. Disease activity (Mayo) scores were assigned prospectively to IBD patients by the physician at the time of endoscopy, and biopsies from 36 UC patients with Mayo scores ranging from 0 to 3 (i.e. normal/inactive disease to severe disease) were coded and sectioned for histological assessment, to assign an inflammatory score, and immunohistochemical (IHC) assessment, to stain for caspases-1, -4 and -5 expression. The data demonstrate a strong correlation (*r*_s_ = 0·636, *P < *0·0001) between the clinical Mayo scores and the histological inflammation scores (Supporting information, Fig. S1), confirming the validity of both scoring systems. Although caspase-1 is expressed at low levels in non-inflamed tissue, it is expressed strongly in both epithelial cells and infiltrating immune cells of the lamina propria in inflamed tissue from patients (Fig. 2a). A comparison of caspase-1 IHC scores revealed a weak correlation with inflammation and endoscopic Mayo scores, revealing that caspase-1 appears to be expressed maximally once inflammation begins and is sustained during moderate and strong inflammation (Fig. 2b,c). In contrast to caspase-1, caspases-4 and -5 expression was restricted to infiltrating immune cells within the lamina propria (Fig. 2d,g), and neither caspases-4 or -5 were detectable within epithelial cells of patients with UC, regardless of the degree of inflammation (Fig. 2d,g). Expression of caspase-4 in the lamina propria correlated clearly with the extent of inflammation (*r*_s_ = 0·502, *P* < 0·0003) (Fig. 2e) and Mayo score (*r*_s_ = 0·504, *P < *0·0002) (Fig. 2f), with maximum caspase-4 expression present in sections with inflammatory and Mayo scores from moderate to strong (scores of 2 and 3). Caspase-5 IHC staining intensity and percentage expression within the lamina propria correlated with inflammatory scores up to moderate levels (*r*_s_ = 0·422, *P < *0·003) (Fig. 2h) and minimal to mild Mayo scores (*r*_s_ = 0·473, *P < *0·0007) (Fig. 2i). An experienced clinical histopathologist (K.S.) identified caspases-4 and -5 IHC positivity in macrophage, neutrophil and lymphocyte populations within the lamina propria of these UC patients.

**Figure 3:**
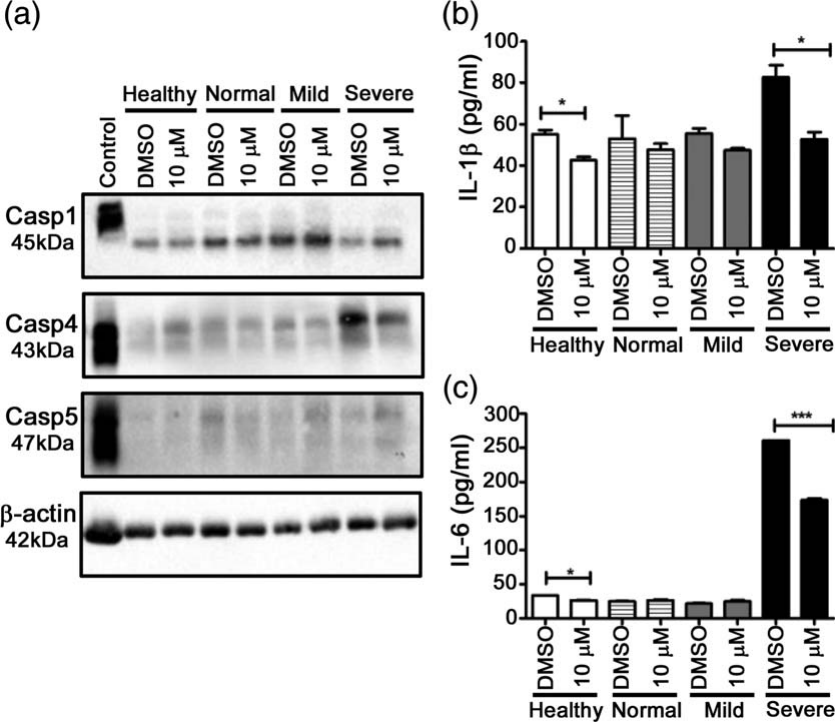
Caspase inhibition attenuates inflammatory cytokine secretion from ulcerative colitis (UC) patient biopsies. Colonic explants biopsy cultures from healthy and UC patients [non-adjacent normal and inflamed tissue (mild and severe)] were incubated with Z-YVAD.fmk [Z-YVAD-FMK (Z-Tyr-Val-Ala-Asp(OMe)-fluoromethylketone)] (10 μM) or vehicle control [dimethylsulphoxide (DMSO)] for 18 h. (a) Caspases-1,-4 and-5 expression levels were detected by Western blotting. Human acute monocytic leukaemia (THP1) cell lysate was used as a positive control for caspase expression. Secreted levels of (b) interleukin (IL)-1β and (c) IL-6 were measured by enzyme-linked immunosorbent assay (ELISA) in culture supernatants from colonic explants. Results are representative of two independent experiments. Data represent mean ± standard error of the mean (s.e.m.); *P < 0·05; ***P < 0·001.

To further address the role of caspases during UC-mediated intestinal inflammation, biopsy tissue from healthy or UC patients with varying degrees of inflammation were incubated *ex vivo* in the presence or absence of the caspase-4 inhibitor, YVAD.fmk. Western blotting revealed elevated caspase-4 expression in UC patients with severe inflammation (Fig. 3a), and ELISAs from biopsy culture supernatants showed that caspase inhibition resulted in reduced levels of IL-1β and IL-6 production from the tissue (Fig. 3b,c). Thus, data from this study reveal a clear involvement for caspase-4 and, to a lesser extent, caspases-5 and -1 in UC-associated inflammation.

**Figure 4:**
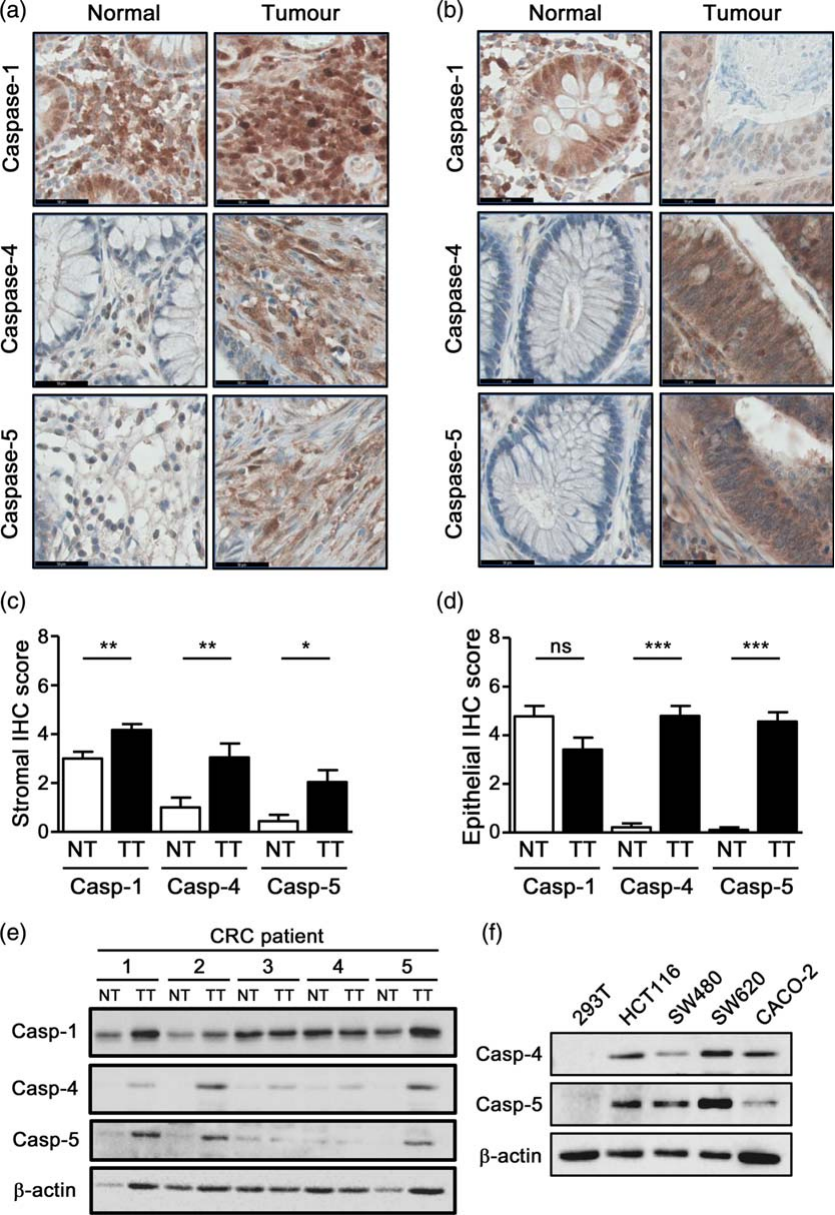
Epithelial expression of caspases-4 and -5 is both specific to, and restricted to, neoplastic colorectal cancer (CRC) tumour tissue. Representative images of caspases-1, -4 and -5 expression patterns in (a) stromal and (b) epithelial regions of adjacent normal (NT) and tumour (TT) CRC patient resection tissue. Defined areas of TT and NT were IHC scored for caspases-1, -4, and -5 expression in (c) stromal; and (d) epithelial layers. Data represent mean ± standard error of the mean (s.e.m.); *P < 0·05; **P < 0·01; ***P < 0·001 (Mann–Whitney *U*-test). Caspase-1 NT (*n* = 18), caspase-1 TT (*n* = 22); caspase-4 NT (*n* = 18), caspase-4 TT (*n = *23); and caspase-5 NT (*n* = 18), caspase-5 TT (*n* = 24). Scale bar = 50 μm. (e) Distal to tumour (non-adjacent) normal (N) and tumour tissue (T) lysates (20 μg) from CRC patient resections were probed by Western blot for caspase-1, -4 and -5. (f) Western blotting for caspase-4 and caspase-5 in lysates (20 μg) from 293T (control), HCT116, SW480, SW620 and CACO2 cells. β-actin was used as a loading control. Results are representative of three independent experiments.

IBD is a risk factor for the development of colorectal cancer (CRC). Having demonstrated the involvement of inflammatory caspases during IBD, we sought to determine whether their expression was also elevated in CRC. Colorectal tumour and adjacent normal tissue samples from CRC patients were stained for expression of caspases-1, -4 and -5 by IHC. The CRC samples, regardless of the stage of tumour progression or pathway of genetic instability (Table1), exhibited similar profiles for each of the inflammatory caspases. Within the lamina propria, caspase-1 was expressed in infiltrating immune cells of tumour tissue and in those of adjacent normal tissue. Interestingly, there was a more marked increase in caspases-4 and -5 expression levels within the lamina propria of tumour tissue compared with adjacent normal tissue, reflecting the enhanced inflammation occurring within the local tumour microenvironment (Fig. 4a,c). Similar to observations from UC patients, caspases-4 and -5 staining was identified independently in macrophage, lymphocyte and plasma cell populations within the lamina propria of CRC patients by an experienced clinical histopathologist (E.W.K.). Inflammatory caspase expression in epithelial cell layers contrasted greatly with the profiles observed in the stroma (Fig. 4b,d). Epithelial cell layers exhibited a striking difference in caspases-4 and -5 expression levels between normal and neoplastic tissue. Similar to our observations from UC patient biopsies, no expression of either caspases-4 or -5 could be detected in adjacent normal tissue; however, high levels of caspase-4 and -5 were present in tumour epithelial cells (Fig. 4b,d). With the exception of one sample (patient 20, Table1), the switch to epithelial expression of caspases-4 and -5 was evident in all malignant tissues examined. To investigate whether the overall increase in caspases-4 and -5 expression could be detected in CRC patient tissue lysates, areas of normal and tumour tissue were examined by Western blot (Fig. 4e). The results show increased caspase-4 expression in malignant homogenates from each of the CRC patients examined, and increased caspase-5 expression was detected in three of the five tumour homogenates examined (Fig. 4e). In support of these observations, examination of a panel of cell lines by Western blotting revealed that all CRC cell lines exhibited robust caspases-4 and -5 expression levels (Fig. 4f). These data suggest that epithelial expression of caspases-4 and -5 may represent biomarkers of colon carcinoma.

**Figure 5:**
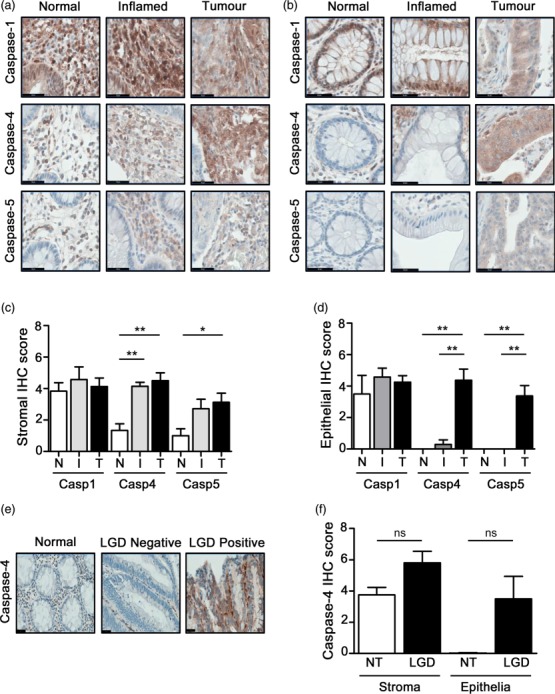
IHC staining of colitis-associated CRC (CAC) patient resection tissue and polyp tissue confirms tumour-specific epithelial expression of caspases-4 and -5. Representative images of caspases-1, -4 and -5 expression patterns in normal, inflamed and tumour (a) stromal; and (b) epithelial tissue from CAC patient biopsies. Defined areas of: normal (N) (*n* = 6); inflamed (I) (*n* = 7); and tumour (T) (*n* = 8) tissue from CAC patient biopsies were immunohistochemistry (IHC) scored for caspases-1, -4 and -5 expression in (c) stromal; and (d) epithelial regions. (e) Representative images of caspase-4 expression in defined areas of normal (N) and low-grade dysplastic (LGD) tissue from colon polyp tissue. (f) Defined areas of N and LGD from colon polyp tissues (*n* = 5) were IHC-scored for stromal and epithelial caspase-4 expression. Data represent mean ± standard error of the mean (s.e.m.); *P < 0·05; *P < 0·01 (Mann–Whitney *U*-test); scale bar = 50 μm.

Current chemoprevention strategies for IBD patients include maintenance anti-inflammatory medications to prevent dysplasia/cancer development, and surveillance colonoscopy to identify and treat early lesions. However, dysplastic lesions associated with IBD-CRC are often difficult to detect and to grade for severity 29. To determine at which point in the inflammation–dysplasia–carcinoma sequence caspases-4 and -5 expression in epithelial cells is up-regulated, a set of UC-associated CRC patient resections were examined in areas of normal, inflamed and tumour tissue for inflammatory caspase expression. Scoring of the lamina propria in tissue from these patients had similar expression profiles to those observed previously in UC patients (Fig. 2) and CRC patients (Fig. 4a,c), with increased expression of caspases-4 and -5 in both inflamed and tumour tissue, while normal tissue displayed high levels of caspase-1, making it less specific to the inflammatory/malignant status of the tissue (Fig. 5a,c). Strikingly, epithelial expression of caspases-4 and -5 were still restricted dramatically to neoplastic tissue, even within areas of severely inflamed tissue (Fig. 5b,d). Areas of normal and inflamed tissue from CRC patient resections all remained completely negative for epithelial caspases-4 and -5 expression (Fig. 5b,d), although a marked increase in caspases-4 and -5 positively stained intraepithelial infiltrating lymphocytes and macrophages was observed in areas of inflamed/dysplastic tissue. In a further attempt to determine the neoplastic stage at which the switch to epithelial expression of caspases-4 and -5 occurs, normal and dysplastic areas of polyp tissue (identified by clinical pathologist E.W.K.) were stained and scored for caspase-4. As observed previously, all areas of normal mucosa were void of epithelial caspase-4 expression. Four of the five dysplastic polyp tissues examined were positive for epithelial caspase-4, while one remained negative (Fig. 5e,f), suggesting that the switch to epithelial expression may occur during low-grade dysplasia. To investigate this hypothesis further, dysplastic CRC tissue was identified in three of the CRC patient resections analysed previously to generate data for Fig. 4. The three dysplastic areas were identified as being low-grade (LGD), high-grade (HGD) and a mix of low- and high-grade (LGD/HGD). Both LGD and HGD were negative for epithelial caspase-4 expression, while the LGD/HGD tissue was epithelial-positive (Supporting information, Fig. S2), highlighting the difficulty associated with determining the stage at which epithelial expression of caspase-4 occurs. Larger patient numbers and mechanistic analysis, in parallel with other neoplastic events, such as the epithelial–mesenchymal transition (EMT), will be required before any real understanding of when, and how, the switch to epithelial expression of caspases-4 and -5 occurs.

These findings reveal that caspases-4 and -5 are discriminators between inflamed and neoplastic tissue, and suggests that there may be significant scope for the detection of epithelial caspases-4 and -5 as determinants of malignancy within colorectal mucosa. The presence of caspases-4 and -5 in epithelial cells could be developed as a complementary marker to histological assessment of dysplasia within colon tissue when predicting CRC risk in patients.

## Discussion

This study demonstrates that inflammatory caspases are expressed highly by infiltrating immune cells in areas of active inflammation in the gastrointestinal tract of IBD patients. Relative expression levels, particularly of caspases-4 and -5, correlate very closely with the level of tissue inflammation and the grade of disease activity in UC patients, suggesting that their enhanced expression has a significant role in disease pathogenesis. The observation showing elevated caspase-4 mRNA levels in healthy colon tissue from IBD patients (Fig. 1c) was not supported by IHC scoring of caspase-4 protein levels in UC patients (Fig. 2e,f); thus, whether basal caspase-4 expression is elevated in healthy IBD mucosa compared with that of healthy, disease-free patients has yet to be clarified.

Similar to observations in IBD patients, we demonstrate high expression levels of caspases-4 and -5 in the stromal compartment of colorectal cancer tissue. Strikingly, we found a dramatic switch in the expression pattern of caspases-4 and -5 in epithelial cells, from no expression in normal or inflamed tissues to strong expression in neoplastic tissue. Although the mechanistic details for these observations are still under way, these findings reveal strong evidence for the involvement of caspases-4 and -5 during colorectal inflammation and carcinoma. As such, this study identifies caspases-4 and -5 as markers of inflammation and the diagnosis of colorectal cancer.

The expression of inflammatory caspases within the stroma of both IBD patients and CRC patients appears to be primarily within infiltrating immune cells, with macrophages, neutrophils, lymphocytes and plasma cells all found to express caspases-4 and -5. A strong correlation between inflammation score and caspases-4 and -5 expression was observed in UC patient biopsies, which supports the hypothesis that caspases-4 and -5 contribute to intestinal inflammation, most probably through activation of the non-canonical inflammasome, leading to increased IL-1β and IL-18 production 24,25. Although evidence for the importance of caspases-4 and -5-mediated non-canonical inflammasome activation during human inflammation is emerging 17,30,31, the majority of studies to date characterizing non-canonical inflammasome activation have been carried out in mice. Recent publications from our laboratory and others have demonstrated a protective role for the non-canonical inflammasome during a murine model of acute colitis 32,33. Here, we provide evidence of caspases-4 and -5-mediated non-canonical inflammasome activation during intestinal inflammation in UC patients.

Inflammatory caspases are under transcriptional regulation 24,34. While caspases-1 and -4 are basally expressed in monocyte (THP-1) and epithelial carcinoma (HT-29) cell lines, human caspase-5 and murine caspase-11 expression is induced in both cell types following stimulation with bacterial lipopolysaccharide or the pleiotrophic cytokine, interferon (IFN)-γ 32,34. Inflammation is an essential driving force in the development of epithelial-originated tumours, and our observation of an increased infiltration of caspases-4 and -5-expressing immune cells to the developing tumour site may serve to establish the tumour inflammatory microenvironment 35. Instead of repressing tumour growth, inflammatory cells can promote tumour growth, particularly during inflammation-associated cancer 36. Another significant observation from this study is the strong expression of caspases-4 and -5 within the neoplastic epithelium of CRC patients. Within tumour areas, stromal staining for caspases-4 and -5 was even stronger than that observed in inflamed tissue, and epithelial cells became strongly positive for caspases-4 and -5, suggesting that inflammatory cells may have a role in inducing the expression of caspases-4 and -5 in colon carcinomas. A recent study using co-transplanted endothelial colony forming and mesenchymal stem cells revealed a role for caspase-4 expression in neovasculogenesis 37. Exposure of epithelial cells, or their precursor intestinal stem cells, to proinflammatory cytokines can induce cancer stem cell (CSC) markers and cause tumour formation *in vivo* 38. This may represent a plausible mechanism for the switch to caspases-4 and -5 expression in neoplastic epithelial cells. Alternatively, there may be different, unrelated mechanisms responsible for caspases-4 and -5 expression in inflammatory cells and epithelial cells, particularly as expression in neoplastic epithelium is observed in both IBD and sporadic CRC tissue. The transcriptional regulation of inflammatory caspases in myeloid and non-myeloid cell types is an area which requires further investigation.

Expression of caspases-4 and -5 within epithelial cells may have a different role than within infiltrating immune cells of the stroma. Previous analysis of the role of nuclear factor-kappa B (NF-κB) in both epithelial and infiltrating myeloid cells during a murine model of colitis-associated CRC revealed that activation of this inflammatory transcription factor in the two different cell types drives carcinogenesis through distinct mechanisms, involving stimulation of proinflammatory cytokines by infiltrating myeloid cells and prevention of intestinal epithelial cell (IEC) death with tumorigenic potential 39. Caspase-4 has been attributed previously with a role in lipopolysaccharide (LPS)-mediated NF-κB activation 40. Thus, caspases-4 and -5 may be responsible for driving tumour initiation by preventing the death of IECs during CRC.

There are no defined clinical features to predict progression from inflammation to low-grade dysplasia, and on to advanced neoplasia. Therefore, histological assessment of biopsy tissue and the identification of dysplasia are relied upon heavily when making decisions regarding the management of IBD and the associated risk of CRC development, and when challenged with the decision of the requirement for colectomy. However, there are a number of issues associated with this current CRC surveillance system: first, the difficulty in identification of true dysplasia *versus* chronic colonic inflammation 41, which explains why there is a high level of inconsistency among pathologists regarding the diagnosis of true dysplasia/neoplasia 42,43. Secondly, patients undergoing regular colonoscopic surveillance can develop CRC without prior dysplasia, and there is no requirement for low-grade dysplasia to progress to high-grade dysplasia before malignancy arises 44,45. Therefore, there is a serious requirement for identifiers of neoplasia which can be assessed in parallel with tissue histology for the identification of CRC. We present the expression of caspases-4 and -5 within intestinal epithelial cells as highly specific biomarkers of colorectal carcinoma, regardless of whether it occurs following IBD or sporadically. The diagnostic relevance of our findings is for inflammation-associated cancer, as distinguishing between neoplastic and chronically inflamed tissue still represents a major challenge for pathologists. Our findings may be relevant for the diagnosis of CRC, as diagnostic tests to assess caspases-4 and -5 expression levels within IECs may be developed in light of these observations. Furthermore, our findings that caspases-4 and -5 contribute to the inflammatory status in IBD patients reveals these two caspases as potential targets for dampening intestinal inflammation.

## Supporting Information

Supporting information: Additional Supporting information may be found in the online version of this article at the publisher's Web site:

**Fig. S1**. Strong correlation (*r*_s_ = 0·636, *P* < 0·0001) between pathological inflammation scores and clinical Mayo scores in ulcerative colitis (UC) patient biopsies. Inflammation scores assigned by a pathologist to UC patient. Biopsies were graphed against Mayo scores assigned by the physician at time of endoscopy. Data represent mean ± standard error of the mean (s.e.m.); **P < *0·05; ***P < *0·01; ****P < *0·001 (Mann–Whitney–*U*-test).

**Fig. S2**. Caspase-4 immunohistochemistry (IHC) staining in dysplastic colorectal cancer (CRC) patient-resected tissue. Images (a) and IHC scores (b) from defined areas of normal, low-grade dysplasia (LGD), high-grade dysplasia (HGD) and LGD/HGD mix; CRC patient-resected colon tissue IHC-stained for caspase-4 (*n* = 1 for each score).
