# Correction to: Altered expression of caspases-4 and -5 during inflammatory bowel disease and colorectal cancer: Diagnostic and therapeutic potential

**DOI:** 10.1093/cei/uxae103

**Published:** 2024-11-28

This is a correction to: B Flood, K Oficjalska, D Laukens, J Fay, A O’Grady, F Caiazza, Z Heetun, K H G Mills, K Sheahan, E J Ryan, G A Doherty, E Kay, E M Creagh, Altered expression of caspases-4 and -5 during inflammatory bowel disease and colorectal cancer: Diagnostic and therapeutic potential, *Clinical and Experimental Immunology*, Volume 181, Issue 1, July 2015, Pages 39–50, https://doi.org/10.1111/cei.12617

An incorrect image representing normal epithelial caspase-4 staining was inadvertently used in Figure 4(b) of the original publication. This was identified by ImageTwin software, referred to on PubPeer (https://pubpeer.com/publications/B13DBCFCC97498E8D150CE855CA3AE). A correct image of caspase-4 IHC stained normal tissue has been included in the modified Figure 4(b), below. The correct image confirms that normal epithelial tissue does not express caspase-4, and does not change the overall findings. The accompanying epithelial IHC scoring (Figure 4(d)) was not affected by the incorrect image used for caspase-4 in original Figure 4(b).


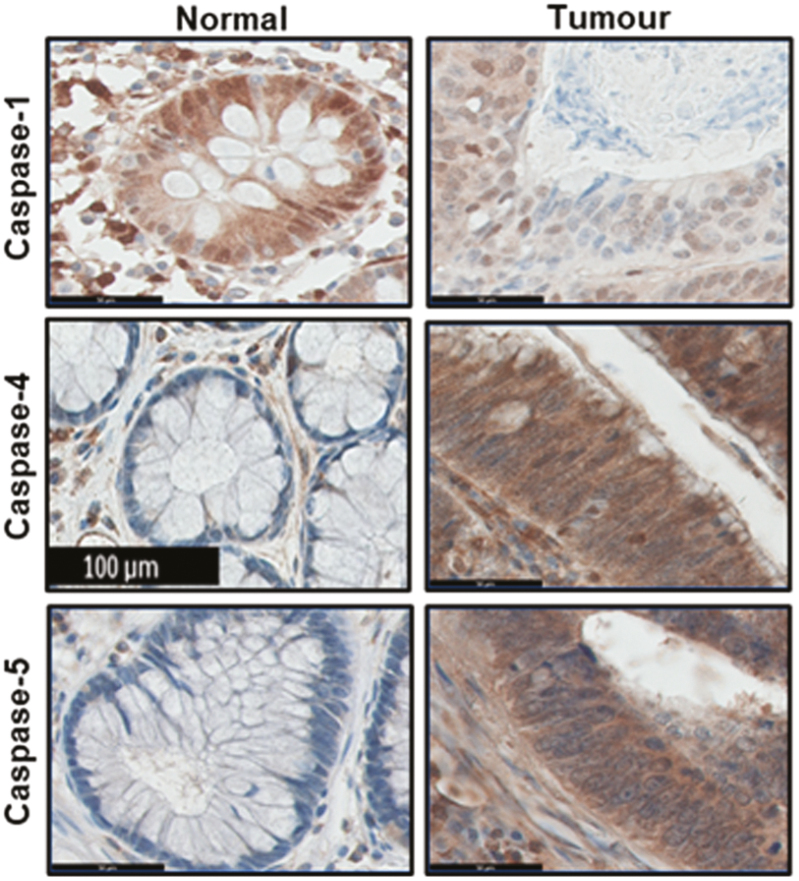


These details have been corrected only in this correction notice to preserve the published version of record.

