# Supplementary figures and images for: Altered expression of caspases-4 and -5 during inflammatory bowel disease and colorectal cancer: Diagnostic and therapeutic potential
Source: Clin Exp Immunol. 2015 May 6;181(1):39–50. doi: 10.1111/cei.12617 (PMC4469154; doi:10.1111/cei.12617)

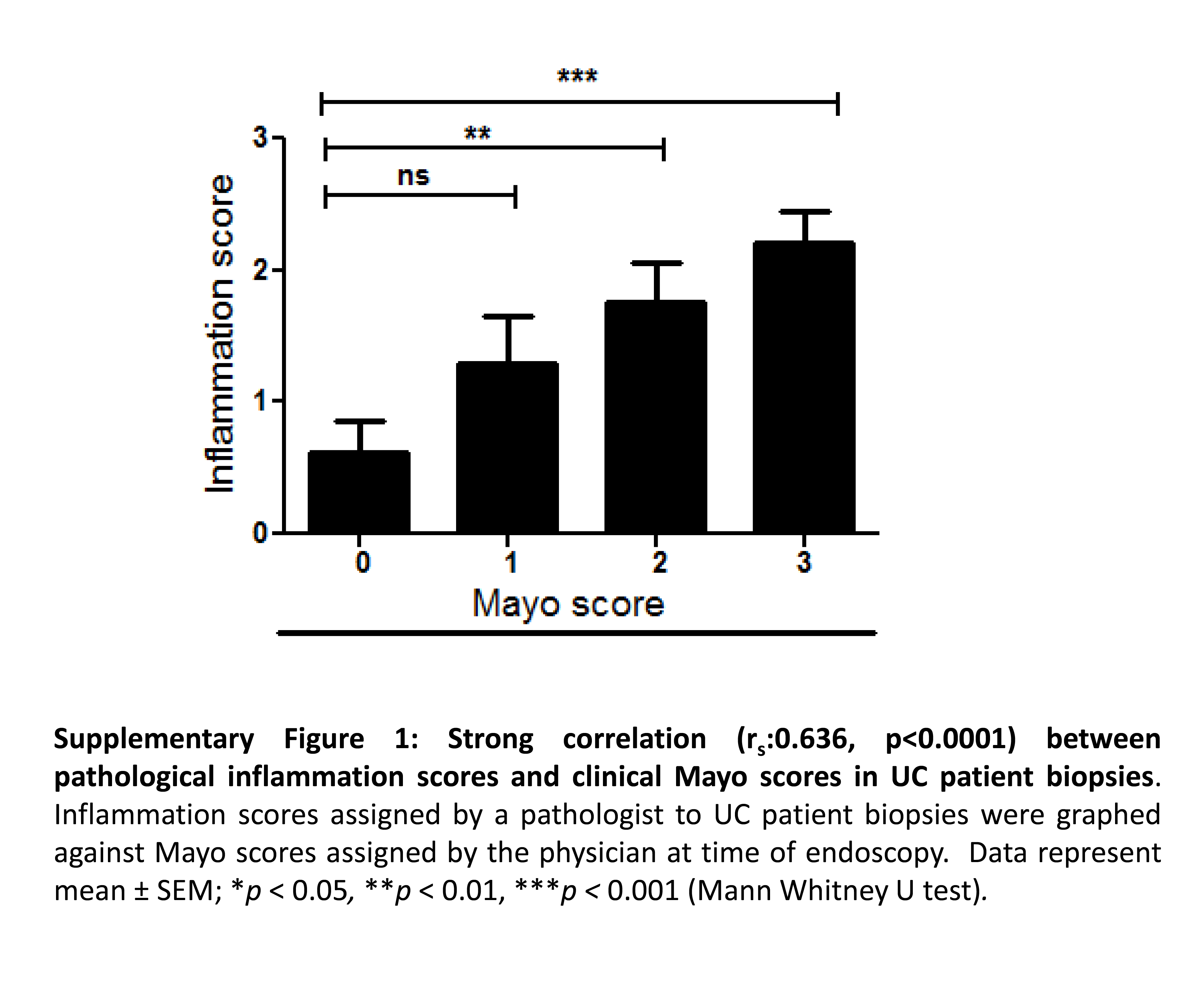

Supplement: Supplementary file 1 [file cei0181-0039-sd1.tif]

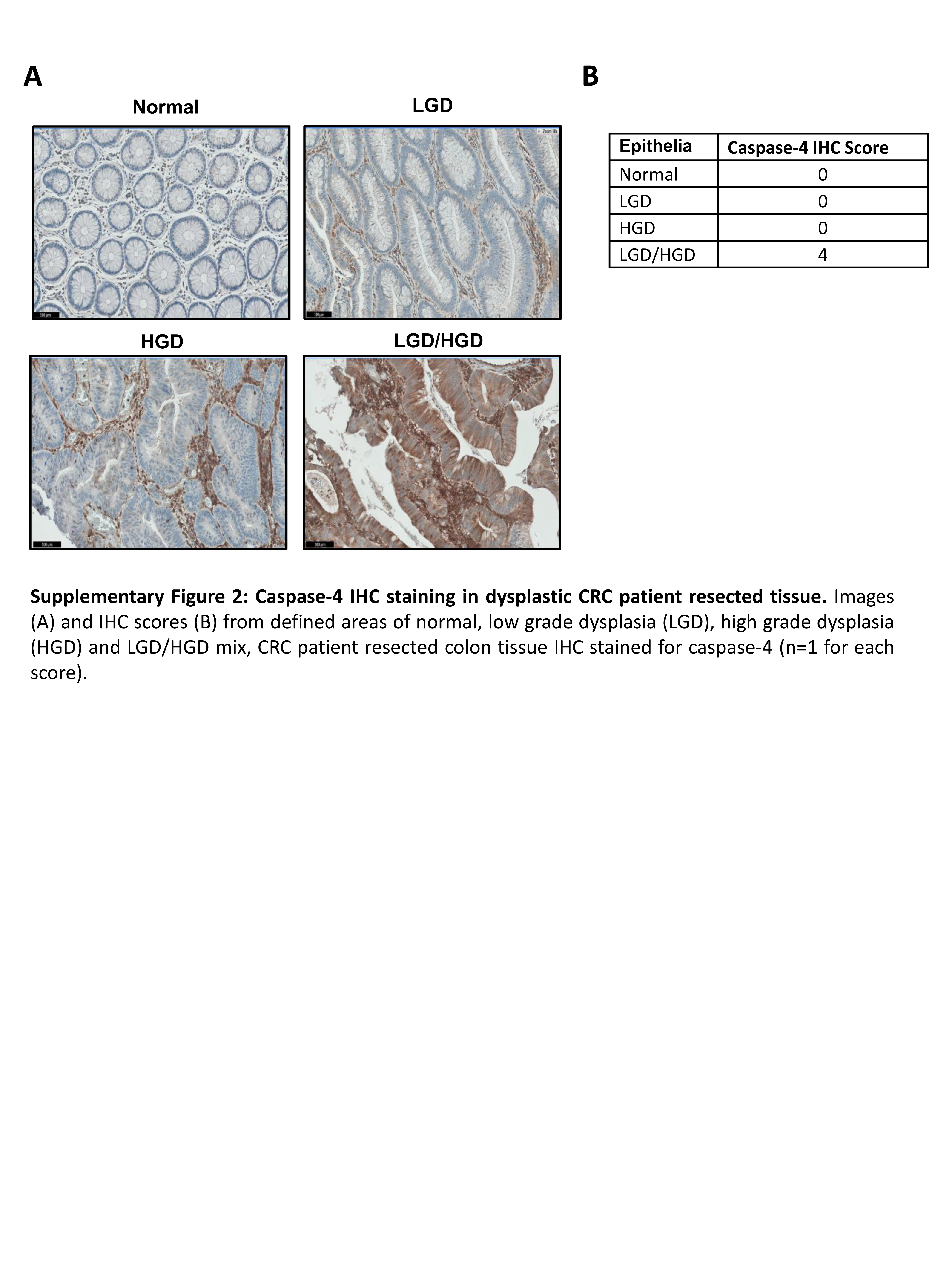

Supplement: Supplementary file 2 [file cei0181-0039-sd2.tif]
